# Finding the Best Match — a Case Study on the (Text-)Feature and Model Choice in Digital Mental Health Interventions

**DOI:** 10.1007/s41666-023-00148-z

**Published:** 2023-09-18

**Authors:** Kirsten Zantvoort, Jonas Scharfenberger, Leif Boß, Dirk Lehr, Burkhardt Funk

**Affiliations:** 1 Institute of Information Systems, Leuphana University, Lüneburg, Germany; 2 Institute of Psychology, Leuphana University, Lüneburg, Germany

**Keywords:** Precision psychiatry, Health care analytics, Machine learning, Natural language processing, E-mental health

## Abstract

With the need for psychological help long exceeding the supply, finding ways of scaling, and better allocating mental health support is a necessity. This paper contributes by investigating how to best predict intervention dropout and failure to allow for a need-based adaptation of treatment. We systematically compare the predictive power of different text representation methods (metadata, TF-IDF, sentiment and topic analysis, and word embeddings) in combination with supplementary numerical inputs (socio-demographic, evaluation, and closed-question data). Additionally, we address the research gap of which ML model types — ranging from linear to sophisticated deep learning models — are best suited for different features and outcome variables. To this end, we analyze nearly 16.000 open-text answers from 849 German-speaking users in a Digital Mental Health Intervention (DMHI) for stress. Our research proves that — contrary to previous findings — there is great promise in using neural network approaches on DMHI text data. We propose a task-specific LSTM-based model architecture to tackle the challenge of long input sequences and thereby demonstrate the potential of word embeddings (AUC scores of up to 0.7) for predictions in DMHIs. Despite the relatively small data set, sequential deep learning models, on average, outperform simpler features such as metadata and bag-of-words approaches when predicting dropout. The conclusion is that user-generated text of the first two sessions carries predictive power regarding patients’ dropout and intervention failure risk. Furthermore, the match between the sophistication of features and models needs to be closely considered to optimize results, and additional non-text features increase prediction results.

**Supplementary Information:**

The online version contains supplementary material available at 10.1007/s41666-023-00148-z.

## Introduction

Estimates suggest that even before 2020, only a third of people affected by mental health problems received the help they needed [1, 2]. This unmet need is accelerated by the psychological aftermath of the COVID-19 crisis, with estimated growth rates in the prevalence of major depression and anxiety disorders of more than 25% [3]. Consequently, offering effective help on a larger scale is of paramount importance for individuals and, considering costs and devastating impacts, for societies as a whole [4].

Digital mental health interventions (DMHIs) help to provide psychological treatment as they are easily accessible, economical, and scalable [5]. DMHIs pursue related goals to face-to-face therapy but are conducted through the means of online education formats. They mainly consist of self-help texts, video or audio manuals, and exercises and can be accessed independently of time and location. DMHIs can be unguided self-help interventions or can include guidance by e-coaches, for example, via calls or messages [6]. Meta-analyses demonstrate their efficacy in treating various mental health problems like stress [7] and stress-related disorders such as depression [8, 9] and anxiety [10]. At the same time, to be effective, a participant must finish at least a certain amount of the intervention to show health benefits [11, 12]. However, it is estimated that in unguided DMHIs, three out of four participants drop out too early. At one in three participants, the odds are better, yet still problematic, in guided DMHIs [13]. Such dropout is a key factor identified for participants’ variance in response rates, causing Gan et al. [12] to call for strategies to help those who struggle. Measures such as e-coaches’ guidance, reminders, and personalization positively influence overall completion rates and health outcomes [12, 14, 15]. However, the extent of guidance necessary differs among individuals and many complete and benefit from interventions with little or none of the usually costly support. Hence, in order to optimally allocate the limited resources and effectively help as many as possible, participants in need of attention must be identified [16].

Machine learning (ML) models can make individual predictions and have previously been used to estimate intervention dropout and failure probabilities [17]. Most of these attempts focus on user journey data, including log-in data and other indicators of online behavior [18–20]. At the same time, human language is the primary tool in psychiatry and psychology [21, 22]. Accordingly, DMHIs often include asynchronous text-driven communication with participants, generally involving (1) open-text intervention exercises and (2) direct communication with e-coaches [23]. Such texts are known to hold valuable information regarding a user’s mental state and intentions that e-coaches can use to best support their participants [24]. Extracting information from these texts is a promising but time-consuming task and thus poses a major challenge with respect to scalability. Natural language processing (NLP) is a field of computer science specifically designed to handle text data. Using NLP methods to automate or augment parts of the e-coaches’ work is a largely unexplored field of research [17]. First advances train ML models on the users’ text to predict binge eating behavior [25] as well as intervention outcomes for social anxiety [26], and depression interventions [27]. NLP methods are ample and differ in both their complexity and their requirements. Obtaining descriptive numbers (e.g., length of the text) and simple counts of words (i.e., bag-of-words approaches) is straightforward from a technical point of view. However, the amount of human decision-making and manual pre-processing is high, and the contextual meaning captured is essentially non-existent. Word embeddings based on neural networks can account for the context of words [28] and have set various NLP prediction performance benchmarks outside of DMHI text data [29]. However, first applications to intervention text data are disenchanting when paired with simple classifiers [25, 27]. With some results worse than random chance, Gogoulou et al. [27] conclude that “the task of predicting treatment outcome based on patient text is very difficult” [27, p. 578]. These results notwithstanding, word embedding features are successfully combined with more complex ML models in the related field of mental health diagnostics [30, 31]. As these conflicting findings show, deciding on a suitable combination of text representation techniques and ML models remains a largely unexplored problem in DMHIs. In addition, the predictive power of newer deep learning models, such as bidirectional encoder representations from transformers (BERT) [29] is yet to be explored in the context of intervention text data. Beyond the issues discussed thus far, Funk et al. [25] point out that the isolated investigation of text data overlooks the likely interaction with non-text features such as the age of participants – a hypothesis supported by several other authors’ findings [26, 32, 33]. Hence, the main motives driving this research are (1) the open question of how to best combine automated text analysis with non-text features to optimize resource allocation in DMHIs, (2) the hypothesis that previous performances of word embeddings in DMHIs are limited by the subsequent classification models used, not the word embeddings themselves, and (3) the proposition that a BERT model pre-trained on a general corpus will have predictive power in the intervention setting as well.

Joining the rising efforts of ML applications and automatization in the health sector [34, 35], we tackle the problem of machine-learning-aided decision-making in E-mental health research. Within this research area, our clinical application is optimizing resource allocation to relieve an overstrained system by identifying those that most need additional support. The findings of our case study on 849 participants allow for the derivation of more concrete hypotheses for the further investigation of empirical generalization [36]. More precisely, our contribution is threefold: First, we systematically compare the predictive power of different text representation methods (i.e., metadata, TF-IDF, topic analysis, sentiment analysis, and word embeddings) in combination with supplementary numerical inputs (socio-demographic, evaluation, and closed-question data) for intervention dropout and failure. We complement related work by investigating which ML model types — ranging from linear to sophisticated deep learning models — are best suited for different features and outcome variables. Second, we account for the relatively long and sequential input texts by designing a task-specific neural network architecture which (in many settings) outperforms existing word embedding approaches on intervention text. Third, we demonstrate the potential of BERT models [29] pre-trained on generic text corpora in dropout prediction. To this end, this paper is structured as follows: we summarize related work (Chapter 2), describe our research approach (Chapter 3), present the text representation techniques (Chapter 4) and ML models (Chapter 5), and thoroughly evaluate different combinations of text representation methods and ML models (Chapter 6). Finally, we discuss the limitations of our study and highlight future research directions (Chapter 7).

## Background

In medical research, the number of ML applications has greatly increased in recent years as they promise improved care, scalability, and cost efficiency [37]. Such improvements are particularly needed in mental health care, where patients often go undiagnosed [38], and require long-time monitoring and care [39]. While many data types (e.g., log-in or questionnaire data) are available [17], text data presents itself as a propitious option in a field that has always primarily relied on language for diagnosis and treatment [21, 25, 40]. Several research branches emerged to leverage text data's vast occurrence in the context of mental health [33]. As their nature, accessibility, and use significantly differ, Becker et al. [40] call for differentiation between research on *pre*-intervention and intervention data. This chapter briefly explains both and outlines related work to derive the research gaps addressed in this study.

### Pre-Intervention Text Data

Pre-intervention data is gathered before and, thus, outside of a clinical intervention. Use cases focus on diagnosing mental health disorders and generating insights. For this purpose, much attention has been placed on social media data [41–44]. These datasets gather users’ natural communication with each other on platforms like Twitter or Reddit. One example are Cohan et al. [31], who tackle a multi-class diagnosis problem on a dataset of 20.406 self-reportedly diagnosed and 335.952 control users’ social media posts. They find that sequential neural network approaches outperform their non-sequential models trained on Term-frequency Inverse Document Frequency (TF-IDF) [45] features in eight out of nine conditions. Yeruva et al. [44] compare insights on obesity and healthy eating — topics related to eating disorders [46] — from 103.609 Tweets versus 6.602 academic abstracts from PubMed. They propose a pipeline to construct social and contextual word embeddings, which produce valuable insights. Wongkoblap, Vadillo & Curcin [47] predict depression diagnoses for 4.169 Twitter users. On the one hand, they compare the dictionary-based Linguistic Inquiry and Word Count (LIWC) tool [48], a language model, topic analysis, and Usr2Vec [49] features paired with logistic regression (LR) or support vector machines (SVMs). On the other hand, they pair word embeddings with a one-dimensional convolutional neural network (CNN), as well as two task-specific (attention-based) neural network architectures. At AUCs of 0.91–0.93, their sequential models outperform their non-sequential models with AUCs of 0.79–0.88. They explain this gap with the information loss non-sequential models suffer when features are aggregated across words. More recent studies in Mental Health diagnostics go one step further by using a more novel pre-trained BERT model [29], which yields good results [35, 50] and thus shows promise for other areas of text data in E-Mental Health research. As much more work exists than can be discussed here, reviews such as [20, 33], or [41] can be referred to for a more detailed picture.

While pre-intervention text data is usually publicly and easily accessible on large scales (e.g., through crawlers), it lacks health labels such as a reliable clinical diagnosis and must depend on self-published information. Further, anonymity and limited information verification options can cause issues with data quality [33, 47]. In summary, pre-intervention text data was produced in a non-clinical setting and primarily generates diagnoses and epidemiological insights.

### Intervention Text Data

In contrast, intervention data comes from a clinical setting designed to help an already diagnosed user. Here, text is produced by health staff (e.g., Electronic Health Records [41, 51]) or by the users themselves [40]. In DMHIs, users primarily produce answers to open-text questions or conversation data with health staff. Because of the controlled setting, high-quality socio-demographic, longitudinal symptom, and user behavior data is usually available. However, gathering intervention data requires resource-intensive steps such as screening, diagnosis, and the assurance of weeks-long (guided) interventions. Consequently, such data points tend to be costly, and data sets stay small [52]. Additionally, access to existing datasets is extremely limited due to privacy concerns [6, 12]. As a result, Shatte, Hutchinson and Teague [17] find that only 1% of studies investigating ML in a mental health setting investigate intervention data, and barely any consider NLP methods. In agreement with these findings, several authors conclude that NLP on intervention data is vastly understudied despite its substantial potential [17, 25, 33, 41].

In mental health interventions, lack of adherence and responsiveness to treatment are major concerns [6, 11, 53]. As shown by Forsell et al. [16], Pedersen et al. [19], and Pihlaja et al. [54], targeted measures such as human guidance can improve upon these problems but, in an already overstrained system, cannot be offered to all participants. Here, supervised ML models provide great value by identifying those users that require additional care and allowing for individually targeted measures [16]. To present a comprehensive picture of previous work of NLP for dropout and intervention failure prediction, we search PubMed with the query (“Natural Language Processing” OR “NLP”) AND (“Psychology” OR “Psychiatry” OR “DMHI*”) AND (“Predict*” OR “Machine Learning”) AND (“Outcome” OR “Dropout” OR “Adherence”). We include papers that used ML models to make individual dropout or outcome predictions based on user-generated open-text data in DMHIs. We then follow the citations in the related work section for more relevant papers. Furthermore, a PubMed search including a similar query with the term “BERT” did not lead to any studies including user-generated intervention data.

Howes et al. [32] predict intervention outcomes based on chat data between therapists and 167 English-speaking users of a depression and anxiety intervention. Simple LR, linear SVMs, and decision tree (DT) models are trained for classification. The authors conclude that a combination of demographic and metadata yields better results than the slightly more sophisticated sentiment and topic analysis. The best-reported f1 measure improves the baseline from 0.57 to 0.7. However, as they point out, they split several messages of one patient between test and training set in their 10-fold cross-validation. With limited patients available, the combination of age, gender, and therapist can already allow a model to identify an individual participant and infer the result from the training example.

Hoogendorn et al. [26] retrieve information about sentiment, topics, writing style, and word usage from German emails written by 69 social anxiety patients, together with meta and demographic data. They investigate (1) averages and (2) trends per person. They choose the 20 features most correlated with their outcome variable — symptom levels at week 12 — mainly covering single words (17), topics (2), and writing style (1). For classification, they train LR, DT, and random forest (RF) models, arguing that these model types give reasonably good and understandable results. While socio-demographic data alone has no predictive value, complementing it with text data up to week six significantly enhances the prediction performance of their RF model (AUC 0.83).

Smink et al. [55] use 770 participants’ first four out of an average of 20 emails written in a DMHI for alcohol abuse to predict dropout. They retrieve word count and LIWC [48] features and combine them with socio-demographic data. The classifiers used are LR, a neural network, XGBoost, and a Mixed Effect RF model. First, they aggregate the features as means across all four emails for the non-sequential models. Second, they input the features per email into their sequential neural network and RF. Hence, while sequential models are included, they only consider the order of emails, not the sequentially of language itself. The winning XGBoost model performs worse than their baseline, leading to the conclusion that they could not associate their simple email text features with intervention dropout.

Funk et al. [25] use 372 participants’ English messages and intervention text snippets to predict binge eating episodes in the next 24 h. A total of 100 of these participants also have the 6-month follow-up health outcome. The authors compare an array of different methods of text representation: metadata, bag-of-words models including topic and sentiment scores, word embeddings, and Part-of-Speech tagging. To predict short-term symptom severity, they train an LR and an RF model, resulting in a maximum AUC of 0.57 for new users. Additionally, they use LASSO regression to determine the best out of their 220 variables for the long-term outcome prediction. None of the 50 element-wise averaged embedding dimensions are among the most informative features.

Gogoulou et al. [27] compare TF-IDF, Word2Vec, FastText [56], and Doc2Vec [57] text representation on Swedish homework reports of 1.986 users of a depression intervention. The three word embeddings are trained in advance on an additional 4.835 users’ texts from other interventions. In their approach, TF-IDF outperforms the word embeddings in almost all settings, and in some cases, the latter even perform worse than the naïve baseline. With a maximum f1 score of 0.69 (baseline of 0.58), they conclude there is a signal in intervention text data regarding outcome prediction, but word embeddings do not serve to extract it. While their paper has the by far largest sample for intervention text data and considers three different methods of word embeddings, it only uses a simple linear classifier. As such, they do not put their focus on leveraging the sequential nature of word embeddings [58].

In conclusion, only one paper investigates the prediction of dropout based on intervention text data, with little success. However, as the authors propose, features other than the two simple ones included should be investigated [55]. For outcome prediction, several studies find that combining text features with non-text features such as socio-demographic data leads to the best results [26, 32, 33]. This results in the first research focus of this paper presented in the introduction; the question of how to best combine text analysis with non-text features to optimize resource allocation in DMHIs. The works so far suggest that simpler text representation features are superior in their predictive performance. However, datasets were almost always smaller than 250 users, and the focus has been on linear and simpler tree-based classifiers. Those papers including more sophisticated models, only used simple features. Thus, the performance of more sophisticated models, such as ensemble methods and deep neural network classifiers in combination with complex features, remains to be investigated in typical DMHI prediction tasks. This leads us to our second research proposition: Previous performance of word embeddings in DMHIs is limited by the subsequent classification models used, not the word embeddings themselves. Further, successful examples from research on pre-intervention data [35, 50] let us arrive at our third proposition for this paper: That a BERT model pre-trained on a general corpus will have predictive power in the intervention setting as well.

## Study Set-Up

This study addresses the gap in existing research by systematically exploring the predictive power of text (i.e., different metadata, TF-IDF, sentiment and topic analysis, Word2Vec and FastText word embeddings) and non-text data types (i.e., socio-demographic and symptom data, evaluation data, and closed-question data) and their interplay with different model types (i.e., LR, SVMs, XGBoost, AdaBoost, LSTMs, and BERT). We investigate these results for intervention dropout and outcome to provide insights into the use of ML methods to optimize resource allocation. The final goal is better outcomes with equal or lower costs [16, 19]. A key focus of this paper is the investigation of the gap between the word embeddings’ theoretical power and the lack of its manifestation when used on intervention text data. To this end, two different word embeddings are trained and then (1) averaged for non-sequential models and (2) used as they are with a sequential model. Furthermore, we employ BERT to make predictions based on the intervention text data, which — to the best of our knowledge — has not yet been investigated. At the same time, Occam’s razor principle suggests that — ceteris paribus — the simplest model is preferable [59]. Because of this, feature extraction methods and models of different sophistication levels are pit against each other in this exploratory study of how to best predict intervention failure and dropout. With 849 participants, the dataset at hand is larger than all but one of the previous works on intervention text.

### Data Description

For our case study, we consider the data of 927 participants from six randomized controlled trials (Table 1) of an internet-based stress management intervention called GET.ON Stress [7]. The training program comprises seven sessions, planned to be held on a weekly schedule. Each session consists of general information, quizzes, audio and video files, downloadable worksheets, and interactive exercises. The interactive exercises are the most important element in each session. Users work through the exercises by reading or listening to short instructions and then writing their answers into text boxes. In subsequent sessions, many of the text inputs are picked up and displayed again to the user by the system. The core stress coping strategies included in the training program are problem-solving [60] and emotion regulation [61]. At the beginning of the program, participants write about their stressors, goals, and motivations. In each subsequent session, the participants are asked to choose pleasant activities, plan to implement them into their lives, and to reflect on how it went in the subsequent session. In the second and third sessions, participants learn a systematic six-step problem-solving method that can be applied to their problems, again reflecting on it in the subsequent sessions. In sessions four to six, participants learn and practice different emotion regulation techniques, such as muscle and breathing relaxation [61]. In the seventh session, participants reflect on their goals for the training and plan how to continue practicing stress coping in the future. Four weeks after completing session seven, an optional booster session eight is provided. Depending on the trial, participants went through the program as a self-help intervention, were able to ask for feedback, or automatically received written feedback by e-coaches after every session. For more detailed information on the set-up of the intervention and each of the studies, please refer to the primary publications cited in Table 1.

**Table 1:** Overview of the intervention studies included in this analysis

Study	German clinical trials register no.	Publication	Level of human support
1	DRKS00004749	Heber et al. [62]	Intensive guidance^a^
2	DRKS00005112	Ebert et al. [63]	Guidance on demand^b^
3	DRKS00005384	Ebert et al. [64]	No guidance^c^
4	DRKS00005687	Nixon et al. [65]	Guidance on demand
5	DRKS00005990	Ebert et al. [66]	Guidance on demand
6	DRKS00005699	Nixon et al. [67]	No guidance

^a^participants receive written feedback (avg. 30 min) after each session; ^b^participants receive feedback on demand; ^c^participants receive technical support only

In this study, intervention dropout is defined as having finished less than the six core sessions out of eight total sessions. Sessions 7 and 8 are not considered core sessions as they do not convey new material but instead serve as a reflection and repetition session, respectively. As such, the dropout definition follows the consensus of operationalizing dropout reported by Donkin et al. [11] and is recommended to use by Gan et al. [12]. The second session is chosen as the point of prediction due to the trade-off between text gathered and time left to intervene [18]. Choosing this prediction point results in 849 German-speaking participants who completed exercises in the first two sessions — 25% of whom are considered dropouts. Intervention failure is defined as an improvement of fewer than 5.16 points on the Perceived Stress Scale (PSS) [68, 69], the primary health outcome metric. This threshold value of 5.16 is based on the reliable change index indicating a clinically meaningful change in symptomatology introduced by Jacobson and Truax [70]. The average baseline PSS score is 25 and, after finishing an average of 6.6 sessions, ends at 17. In total, 37% of users considered are intervention failures. A total of 40 participants did not fill out the PSS questionnaire after finishing the intervention and, therefore, cannot be considered for intervention failure prediction. Losing many data rows because participants did not fill out the final symptom questionnaire is a common problem when predicting intervention outcome. For example, Gogoulou et al. [27] disregard 38% of their participants because their low adherence prevents the calculation of the target features. Attempting to predict the 6-month follow-up, Funk et al. [25] even lose 73% of their data. In this dataset, from those with unknown outcomes, 85% dropped out. Excluding these participants runs the risk of ignoring those most in need of additional support. Therefore, we provide insights into both, dropout (keeping more participants) and intervention failure (the more exact outcome measure) predictions.

### Non-Text Data

Related work suggests that a combination of text and non-text features is most promising when retrieving information about a user’s mental state [23]. Thus, unsurprisingly, a myriad of the above-mentioned studies includes non-text variables in their analysis. We train benchmark models on each of the non-text and text feature types by themselves and then combine them to be able to differentiate between individual, and interaction effects.

*Baseline variables* such as socio-demographics or symptom data have been thoroughly investigated in terms of their predictive power for dropout and intervention failure, howbeit with limited consensus in results (e.g., [71, 72]). We include these variables in our analysis based on the assumption that ways of expressing oneself are dependent on users’ characteristics such as age and gender [23]. Asking users to fill out a baseline questionnaire before starting the intervention is common, as seen in the related work section. Our eleven socio-demographic variables cover different information about the participants’ age, gender, educational background (2 features), occupation (5), and family status (2). 77 participants did not indicate their income level, they are accounted for in an additional feature. The descriptive statistics and data types of all included socio-demographic features can be found in the supplementary material 1. The majority of participants identify as female (78%), hold a college degree (60%) and are on average 42 years old, where the age distribution is bimodal with two peaks around 30 and 50 years. In addition to the socio-demographic variables, five symptom-related variables provide the baseline PSS subscores of *Helplessness* and *Self-Efficacy* [69], and carry information about previous experiences with training and therapy (3 features). The mean values of the PSS subscores before the intervention are 16 and 9, respectively. The aforementioned variables are supplemented by the intervention support level and an indicator of whether the user found out about the intervention via their health insurance company.

*Evaluation data* providing information on the user’s attitude towards the intervention can easily be argued to be an evident factor for their intention to continue it. Therefore, this data proposes a promising alternative to the resource-intensive process of text-analysis. At the same time, it requires an additional questionnaire after each session, hence straining the limited user attention available. To investigate this trade-off, it will be included in the analysis. The users evaluate the (1) easiness and (2) usefulness of each session on a scale of 1 (very useful/very easy) to 5 (not useful at all/very difficult). Furthermore, the users were asked to estimate the time they needed to complete the respective session on a rating scale from 1 (less than 30 min) to 4 (more than 90 min). On average, users rate the easiness with 2.3, the usefulness at 1.8 and the time required between 30 and 90 min. Furthermore, users have the chance to articulate well-liked and improvable aspects of each session in an open-text format. For the text representation, we append this text to the rest of the user’s generated text of the corresponding session. In total, 735 participants answered the evaluation questions for at least one of the first two sessions, and missing values are accounted for in an additional feature.

*Closed question data* is structured data in the form of questionnaire items that have a limited set of pre-defined answer options, which Cook et al. [73] found to have better performance than open-text questions when predicting suicidal intentions. Such closed questions are often inherent in the intervention design and are easier to handle than unstructured text data from a technical standpoint. Exemplary impressions of how the users saw such questions can be found in the supplementary material 2. In our dataset, three closed-form intervention exercises sum up to an additional 13.298 user entries. These questions address the perceived stress levels, the percentage of successfully implemented goals from the previous session, and the intended day of finishing the upcoming session. We extract the relevant numbers and — depending on the nature of the question — include them as they are or aggregate them (i.e., sums, averages, or counts). We fill missing values with 0 s and create additional features indicating missing values.

## Text Representation

In total, the 849 users produced 61.290 open-text answers to intervention exercises and another 3.647 answers to the open-text evaluation questions. Given the point in time of the prediction, only the text from sessions one and two are used. This leaves 15.773 entries, 1.597 of which are open-text evaluation answers. As a first step, 1.064 entries that do not contain any relevant information (e.g., “xxx”, “…”, “-”) are deleted, which are found via the investigation of the answers with less than five characters. A feature counting the number of such entries is included in the simple metadata. Since text representation techniques typically cannot handle numbers well [25], digits are replaced by ‘#’. Second, we scrape a list of commonly used German abbreviations and manually adjust and supplement them to better fit the context of this intervention. The abbreviations are replaced with their long-form, and special characters, as well as smileys, are deleted. A spell check based on the Hunspell package is tried but does not increase cross-validation scores and, therefore, is not used in the final results. Third, we lemmatize the participants’ text using the Python library SpaCy. As upper-case letters carry significant meaning in German [74], the texts are only lower-cased after lemmatization. Since bag-of-words methods usually benefit from lemmatized texts [74], while neural network approaches are not expected to [75], we keep both. Lastly, we aggregate the text per user and session, resulting in a concatenated string of all user text inputs that can be used as-is or be further aggregated across sessions 1 and 2.

### Metadata

Especially when thinking about dropout as the binary manifestation of engagement, the effort invested in the exercises is a promising candidate for its prediction [5]. Assuming that a longer answer to a given task requires more effort than a short one, the arguably most straightforward measure is the length of the answer. Hence, we create a *simple metadata* representation of the participants’ texts by measuring the word and character count. An average intervention text in sessions 1 and 2 together contain 617 words in 4.105 characters and an additional 48 words in 313 characters for the evaluation questions. To account for the participants’ willingness to answer the intervention questions, a feature counting the number of *useless* (defined as above) entries is added. Additionally, the usage of upper cases, exclamation, question marks, and positively or negatively connoted smileys are counted before they are deleted in the text cleaning. The *advanced metadata* is based on Ewbank et al.’s [51] finding that different therapeutic intentions and topics have different impacts on outcomes in face-to-face intervention. In sessions 1 and 2, tasks aim to gather information about the user’s motivation and build skills in problem-solving, stress analysis, behavior reflection, and behavioral planning. Thus, all text snippets are categorized, and text lengths per category are retrieved to investigate whether this additional information can improve predictions.

### Bag-of-Words

Bag-of-words approaches count the occurrences of each word in a document (i.e., intervention answers) in an attempt to extract similarities or differences in texts. A popular bag-of-words method is *Term Frequency-Inverse Document Frequency* (TF-IDF) [45]. The word occurrence count is rescaled based on the relative occurrence of all documents. The scikit-learn TF-IDF vectorizer is used on the word level, considering uni- and bigrams to produce the vector per participant. This approach results in a very large and highly sparse matrix; both attributes that many ML models cannot handle well. To reduce the size of the matrix, features used by more than 70% of documents are discarded, as they are assumed to be stop words. In order to keep fewer features than data points [25], the number of TF-IDF features kept is determined by the number of users minus the number of additional non-text features. In two additional steps, sentiment and topic analysis are used to reduce the matrix dimensionality by grouping similar words. *Sentiment properties* of polarity and subjectivity are retrieved per text snippet to extract variation in sentiments depending on the exercise (e.g., “What stressed you today?” vs “What makes you feel good”). The German version of the text blob package - a rule-based approach — is used on the lemmatized text, as per the recommendation of Fehle, Schmidt and Wolff [74]. Sentiment polarity is recorded on a scale from [-1,1], with the minimum indicating a negative and the maximum indicating a positive connotation. In addition, the subjectivity variable indicates the level of opinion, emotions, or judgments between 0 (objective) and 1 (subjective). As both the average sentiment and the range of sentiment are considered valuable information [25], the mean, max, and minimum scores across sessions are included as features. Another way of reducing the dimensions is *Latent Dirichlet Allocation (LDA),* which tries to identify latent topics in the documents. LDA assumes that a document touches upon different topics operationalized by a list of common words associated with each topic [76]. Considering the number of relatively small entries and the likely tendency that similar exercises produce similar answers, this step is done on the already aggregated text, and the number of topics considered is set to 10 [25]. The topic model is calculated on the training data corpus only and then applied to the test data text.

### Embeddings

Based on the assumption that similar words appear in similar contexts, word embeddings attempt to analyze word co-occurrences and represent each word by *n*-dimensional vectors of real numbers. Thus, words used in akin contexts tend to be mapped to vectors with small distances. Word2Vec [58] and FastText [56] are frequently used word embedding techniques based on neural networks. Word2Vec offers two different network architectures to learn word representations by (1) predicting a current word based on its surrounding words (CBOW) or (2) predicting the surrounding words based on a current word (Skip-gram). While the learned representations of the words in the training corpus are mostly meaningful, unseen words cause difficulties. In order to find a vector representation of these words, a fraction of rare words is typically mapped to an out-of-vocabulary (OOV) token during training allowing unseen words to be mapped to this generic OOV vector. FastText [56] is an extension of Word2Vec, which tries to tackle the problem of unseen words by building embeddings for each word in the corpus as well as the n-grams each word consists of. Hence, word vectors for unseen words can be generated based on the n-grams in a more meaningful way. Both word embeddings can be trained from scratch on custom datasets or word embeddings pre-trained on large text corpora in languages (e.g., Wikipedia or News articles) can be used. Since the text produced by the study participants is different in its structure from generic corpora, we follow related work [25, 27] and train the word embeddings on an extended dataset using the Gensim library. To enhance our small training dataset, we also use the texts generated by control group users and train the word embeddings at the sentence level. We treat the vector dimension *n* and the model architecture (i.e., CBOW or Skip-gram) as hyperparameters which are optimized during the training of our recurrent neural network (Section 5.2). To compare the sequential approach to results from related work [25, 27], we process the generated word embeddings by calculating the element-wise averages of every participant’s text and use these averaged word embeddings as inputs for non-sequential models (Section 5.1).

## Machine Learning Models

In the following, we present the different ML models that are trained to predict dropout and intervention failure. To match the complexity of the text representation methods, we consider three different model categories: (1) traditional ML models for non-sequential data, (2) deep learning models for sequential data, and (3) advanced pre-trained transformer-based models. While non-text features, meta-data, bag-of-words, and averaged word embeddings are combined with traditional ML models, we extend related work in this field by additionally maintaining the sequential nature of text by training recurrent neural networks as well as a BERT classification model. We set apart a hold-out test set (20% of the participants) beforehand to evaluate the models’ out-of-sample performance (Chapter 6).

### ML Models for Non-Sequential Data

We use four different classification models: LR, SVMs, AdaBoost, and XGBoost. The corresponding model hyperparameters are optimized in a fivefold cross-validation (CV), where each hyperparameter space is defined by initially choosing small intervals around the default values and incrementally considering adjustments if the boundaries perform best in the CV. For each data input (i.e., combinations of text representations and supplementary numerical inputs), one final model, chosen based on the CV scores, is trained on the entire training data, and evaluated on the hold-out test data. To account for the class imbalance in the dropout data, we create synthetic data of the minority classes by using SMOTE oversampling [77]. The sampling ratio is treated as a hyperparameter for all four models and is optimized during the CV.

*Logistic regression* as a linear model for binary classification is very popular due to its fast training times, good explainability, and reasonably good results. In light of the dataset size, the liblinear solver is chosen. Given the partially high number of predictors, L_1_ or L_2_ regularization are optimized as a hyperparameter in the CV, together with the respective penalization strength (0.01–10). *Support* *vector machines* classify by drawing decision boundaries between classes. SVMs can either use the feature space as is or use a non-linear kernel to map it into a higher dimensional space to make classes linearly separatable [78]. The use of a linear or a radial basis function kernel is optimized as a hyperparameter, each with their own set of regularization parameters (C: 0.1–1000, gamma: 0.001–1) to balance over- and under-fitting. For both, LR and SVMs, a scaler is added to the ML pipeline. *XGBoost* is a fast and efficient implementation of a Gradient Boosting Tree that also allows for the regularization of features and thus avoids overfitting on smaller datasets [79]. As the XGBoost classifier has many non-trivial hyperparameters, Bayesian Search CV is used to allow a less computationally expensive grid search [80]. To constrain the architecture of the trees, the max. depth (3–5), and the minimum weight of a child (0.5–1) are optimized. Further measures against overfitting are the percentage of rows (0.5–1), and columns (0.5–1) used to build each tree, as well as the regularization parameters gamma (0/1) and lambda (1/2). The number of estimators (50–1000) is also investigated with the learning rate for each step (0.01–0.5). *AdaBoost* classifiers leverage the advantages of ensemble learning by combining a variety of weak learners to achieve better predictions [81]. The number of estimators (3–2000) used stands in a trade-off to the learning rate (0.001–2) — the weight given to each estimator — because of which these are optimized together. We implement our models in Python using the Scikit-learn and xgboost libraries. The non-sequential models can be trained on a standard laptop, and training times partially depend on the number of features. Including grid search, LR and SVMs usually need mere seconds while the AdaBoost model, on average, takes several minutes. Training times are the longest for the XGBoost models, where iterating through the entire hyperparameter space often takes longer than for the AdaBoost model, despite the use of Bayesian Search CV. Including the large number of TF-IDF features implies the longest training times at one or two hours each for the Ensemble models.

### Recurrent Neural Network

Related work in this field demonstrates the inferior performance of word embeddings when element-wise averaged and used as inputs for models from the previous section [25]. Due to the relatively long input sequences in the second session (on average 370 words respectively 392 with evaluation texts), we assume that a carefully designed recurrent neural network can better leverage the potential of word embeddings than averaged word vectors and thus possibly achieve better results on our two classification tasks. To avoid enlarging the input sequence length further, we do not include text generated in the first session.

**Fig. 1:**
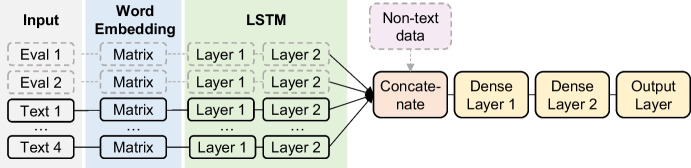
Task-specific LSTM-based model architecture

A naïve bidirectional LSTM-based [82, 83] model architecture, which consists of one input containing all text inputs of a given participant, barely achieves baseline performance on our validation set. This may be grounded in challenges arising from these long input sequences. Therefore, we decide to design a more sophisticated, task-specific model (Fig. 1) for our problem. The core model has four different blocks which aim to encode the participants’ texts with respect to one of the four categories used in the second session — problem solving, reflection, stress analysis, and behavioral planning — and thus naturally reduces the input sequences’ lengths. Each block consists of an input layer, an embedding layer (i.e., our pre-trained word embedding matrix), and two bidirectional LSTM layers. All outputs from the last bidirectional LSTM layers are concatenated and passed to a fully-connected neural network with dropout. Adding a further bidirectional LSTM layer after concatenation does not improve performance on our validation set. We consider the embedding dimension (FastText: 10, 25, 50, 100; Word2Vec: 25, 50, 100, 300), input sequence length (30, 50, 100, 200 words), number of units per LSTM layer (first layer: 0, 16, 32; second layer: 16, 32), number of neurons per dense layer (16, 32), and the dropout rates (0.1, 0.2) as hyperparameters which are optimized during training. If further text inputs are considered (i.e., evaluation texts), we extend our core model by two blocks processing the two different evaluation categories (i.e., feedback about liked contents and suggested improvements). If numerical inputs are considered (i.e., demographical data, numerical evaluation data, or extracted numbers from text), we extend our core model by another input layer which is normalized and directly passed to the concatenation layer. We try to account for the imbalanced class distribution by using a weighted binary cross-entropy loss function. The class weights are considered hyperparameters which are optimized during training. The Adam optimizer is used to train this network architecture where the learning rate (0.01, 0.001, 0.0005) yields the final hyperparameter. To tune all hyperparameters, we use 20% of the training data as a validation set and re-train our tuned models on the entire training set for 25 epochs with early stopping. Since the performance does not increase when fine-tuning the embedding layers, we freeze the embedding weights and only train the remaining weights of the network. The network is implemented in TensorFlow, and the hyperparameter tuning is executed on an Nvidia Tesla P100, which takes approximately six hours for each of the four different data inputs.

### BERT

To represent the more complex recent transformer model architectures, we investigate the prominent “bidirectional encoder representations from transformers” (BERT) model [29] to predict dropout and intervention failure. In contrast to the previous approaches of separating the steps of text representation and training classification models, the BERT model combines these tasks. While training BERT from scratch requires a substantial amount of data, BERT models pre-trained on large datasets can be leveraged and are easily adaptable to new NLP tasks. On NLP benchmark tasks, pre-trained BERT models that are fine-tuned on custom datasets achieve better results than carefully crafted task-specific model architectures [19]. Therefore, we also follow this approach to both maintain the sequential structure of the texts and to reduce the manual effort in designing an appropriate architecture.

We build our classification model based on the BERT model pre-trained on three large German datasets (“bert-base-german-cased” from Huggingface’s model repository) and fine-tune it on our dataset. To adapt this model to our two classification tasks, we slightly modify the model architecture: we use the 768-dimensional representation vector produced by the BERT model and feed it into a new classification head consisting of two hidden layers and a sigmoid output layer. When considering additional numerical inputs (i.e., baseline, evaluation, or closed question variables), we concatenate the 768-dimensional vector with the supplementary variables. The design of the classification head is optimized during training where the number of neurons per hidden layer (16, 32, 64) and the dropout rate (0.1, 0.2) are considered hyperparameters. Despite BERT’s ability to handle input sequences up to 512 words, we only consider shorter lengths (64 and 128 words) due to the required computational resources. To compensate for the class imbalances, we make use of a weighted binary cross-entropy loss function and treat the class weights as additional hyperparameters. The aforementioned hyperparameters and the learning rate ($5\cdot {10}^{-4}$, ${10}^{-5}$, $5\cdot {10}^{-5}$, ${10}^{-6}$) of the Adam optimizer are optimized during training using a validation set of 20% of the training data. The final model is trained on the entire training data for 20 epochs with early stopping.

## Results

We evaluate our final models on the test set of 170 (dropout) and 163 (intervention failure) participants. Although our test set is large when compared to most related work, this size still implies the risk of unrepresentative results. Since the area under the receiver operating characteristics (AUC) accounts for class imbalance [84] and thus eases the comparison of results of the two classification tasks, we choose this evaluation metric [85]. The two result tables for intervention failure (Table 2) and intervention dropout (Table 3) summarize the AUC scores on our test set, where columns represent different text representation methods and rows define supplementary non-text features. The benchmark model (BM) column provides a reference score trained exclusively on the corresponding numerical features. To identify the most predictive features, we calculate SHAP values [86] or use included feature importance measures for the non-sequential models.

**Table 2:** Result table intervention failure prediction

AUC	BM	Sim. MD	Adv. MD	TF-IDF	Sentiment	LDA	W2V Avg	FT Avg	W2V NN	FT NN	BERT
Pure Text	0.500	0.546	0.588	0.545	0.649	0.550	0.500	0.522	0.605	0.553	0.550
Baseline	0.688	0.687	0.710	0.687	0.719	0.687	0.687	0.687	0.635	0.617	0.581
Eval	0.624	0.623	0.631	0.520	0.649	0.629	0.615	0.596	0.676	0.592	0.591
Closed-Q	0.529	0.524	0.578	0.554	0.577	0.534	0.508	0.530	0.572	0.623	0.564
Average	**0.614**	**0.595**	**0.627**	**0.577**	**0.649**	**0.600**	**0.578**	**0.584**	**0.622**	**0.596**	**0.572**

*BM*, baseline; *Sim./ Adv. MD*, simple or advanced metadata; *W2V*, Word2Vec; *FT*, FastText; *NN*, task-specific neural network; *Eval*., evaluation data; *Closed-Q*, closed questions

**Table 3:** Result table intervention dropout prediction

AUC	BM	Sim. MD	Adv. MD	TF-IDF	Sentiment	LDA	W2V Avg	FT Avg	W2V NN	FT NN	BERT
Pure Text	0.500	0.651	0.541	0.626	0.559	0.547	0.531	0.610	0.644	0.630	0.618
Baseline	0.596	0.614	0.633	0.642	0.588	0.617	0.662	0.551	0.696	0.645	0.646
Eval	0.649	0.686	0.592	0.651	0.636	0.643	0.639	0.601	0.636	0.656	0.668
Closed-Q	0.584	0.554	0.563	0.652	0.599	0.606	0.575	0.632	0.639	0.663	0.668
Average	**0.610**	**0.626**	**0.582**	**0.643**	**0.596**	**0.603**	**0.610**	**0.607**	**0.654**	**0.649**	**0.650**

### Intervention Failure

Exclusively considering *text variables*, sentiment analysis (AUC of 0.65) outperforms the other text representation techniques on our test set. Other methods, such as Word2Vec combined with our LSTM architecture and advanced metadata with an LR model, achieve solid results (0.61 resp. 0.59) as well. While averaged word vectors combined with boosting classifiers perform very poorly (0.50–0.52), leveraging the sequential nature of the texts by using deep learning architectures yields benefits (AUC 0.55–0.61). Thus, also performing equally or better than TF-IDF features with an AdaBoost model (0.55).

Our benchmark model (BM) trained on the numerical *baseline data* achieves an AUC score of 0.69 and, hence, is not outperformed by the vast majority of text representation techniques. This is most likely due to the initial PSS subscores included in the baseline data, which are expected to be important variables in predicting intervention failure [16]. While the additional baseline variables increase the performance of all text representation methods (compared to text-only models), only advanced meta-data and sentiment analysis (both combined with LR) achieve better AUC scores (0.71 and 0.72) than our baseline benchmark. In both cases, age is the most important feature, followed by the baseline PSS subscores and income category. While PSS subscores are among the five most important variables of our benchmark model as well, age and income are not, which possibly indicates a moderating function for text features. The deep learning approaches are the only approaches that do not benefit from adding baseline data and perform worse than the averaged word vectors combined with LR. Similar to baseline data, additional *evaluation data* (both textual and numerical) enhances the performance of nearly all representations. Besides the winning task-specific Word2Vec LSTM architecture (0.68), advanced meta-data, sentiment analysis, and LDA (all using LR) achieve better results than the evaluation data benchmark by itself (0.62). TF-IDF, averaged word vector, and the remaining deep learning approaches cannot attain the benchmark scores, suggesting that more variables can have a harmful effect on the information-to-noise ratio. *Closed-question data* adds little value and, in some cases, even decreases the model performance when compared to text-only results. Only the task-specific FastText LSTM model leverages this additional information and achieves an AUC result of 0.62. While this clearly outperforms the benchmark (0.53) as well as the averaged word vectors on this task, various other approaches on different data inputs achieve better results.

To predict intervention failure, baseline data containing initial PSS subscores clearly benefits the models’ performances. On our test set, sentiment analysis and advanced meta-data approaches yield solid results which perform better than benchmark models and other approaches considered. On average, advanced metadata (0.63) performs slightly better than simple metadata (0.60), offering evidence that the nature of the exercise done matters for the intervention outcome. Analyzing the model coefficients of our advanced metadata reveals that the largest coefficients are assigned to the text length of tasks concerning problem reflection, behavioral planning, and motivation. Since this model aims to predict rather than to explain, further research is necessary to investigate the causality. Among the two best-performing non-sequential approaches, LR and SVM are most frequently chosen (6 out of 8). BERT, TF-IDF (primarily used with boosting classifiers), and averaged word embeddings often perform below our benchmark. Although we demonstrate, on our test data, that word embeddings combined with our task-specific architecture often outperform the averaged word vector approaches, the deep learning models fail to achieve benchmark scores in many cases.

### Dropout

Despite the theoretically assumed interrelation between dropout and intervention failure [11, 12, 87], well-performing text representation approaches and ML models differ significantly on our dataset. While TF-IDF and the deep learning approaches perform poorly in many settings when predicting intervention failure, these approaches, as well as the simple meta-data approach, dominate the results for dropout prediction. On pure *text data*, simple meta-data combined with a non-linear kernel SVM classifier yield the best AUC score (0.65), closely followed by TF-IDF combined with XGBoost (0.63) as well as the Word2Vec (0.64) and FastText (0.63) task-specific LSTM models. Word embeddings combined with our LSTM architecture increase performance in comparison to averaged word embeddings and an SVM classifier (0.53 and 0.61). Previously well-performing approaches such as advanced meta-data and sentiment analysis score mediocre results (0.54 resp. 0.56) on the task of dropout prediction. Akin to the intervention failure prediction, model performances’ generally benefit from additional *baseline and evaluation variables*. Yet, for dropout prediction, evaluation data has a stronger impact, supporting the hypothesis that a participant’s opinion on the intervention is a good predictor for discontinuation. The task-specific LSTM-based approach on the Word2Vec embeddings scores the best results (0.70) when using additional baseline variables, and other deep learning approaches also perform well (0.65) in this setting. TF-IDF features used with LR likewise achieve an AUC score (0.64) well above the benchmark (0.60) on this task. Simple meta-data combined with LR (0.69) and our fine-tuned BERT model (0.67) yield the best results when harnessing supplementary evaluation data. SHAP values, calculated for the evaluation benchmark and simple meta-data model, suggest that the number of useless entries, the session’s perceived usefulness, and time adequacy are the most important features in this setting. Our FastText approach slightly surpasses the benchmark of 0.65 on the evaluation data. Using additional *closed-question data* mostly enhances the performance. BERT (0.67), FastText (0.66), Word2Vec (0.64), and SVM trained on TF-IDF features (0.65) clearly outperform the benchmark model (0.58). Averaged FastText (0.63) features combined with SVM achieve solid results, however, they cannot reach the results of our LSTM architecture.

In most cases adding non-text data increases the model performance, most evidently in the case of evaluation data. The most basic approach considered (simple meta-data) outperforms all other approaches when working on pure text data as well as in combination with evaluation data. Thus, a participant’s’ attitude in combination with how much they write is an easily attainable and well-performing prediction set-up. Among the non-neural approaches, at nine times, SVMs with the non-linear kernel are the most commonly chosen classifiers, with an additional two wins for linear SVMs. At six or seven each, LR, AdaBoost, and XGBoost do not differ much in how often they were chosen. The more sophisticated approaches (TF-IDF, word embeddings in combination with task-specific LSTM architectures, and BERT) constantly achieve good results, and on average yield the best AUC scores on our test set. We notice a pattern in the embedding dimension and input sequence length hyperparameters: the most prominent embedding dimension among Word2Vec models is 25 with a maximum input sequence length of 100, whereas FastText models prefer shorter sequences of 50 words and embedding dimensions of 10 or 25. These findings also hold when predicting intervention failure, thus indicating the need to treat these numbers as hyperparameters instead of choosing default values. Furthermore, most models do not benefit from the second (optional) LSTM layer, which points towards an overwhelming model complexity considering our dataset size.

## Discussion of Clinical Usefulness

As discussed by several authors such as Olczak et al. [85], Cabitza and Campagner [88], and Scott, Cater and Coiera [89], prediction performance metrics are only one subdimension when evaluating ML models in health care settings. Therefore, we use the ten questions proposed by Scott, Cater and Coiera [89] to summarize and evaluate the prospective clinical value of the proposed winning models.

**Fig. 2:**
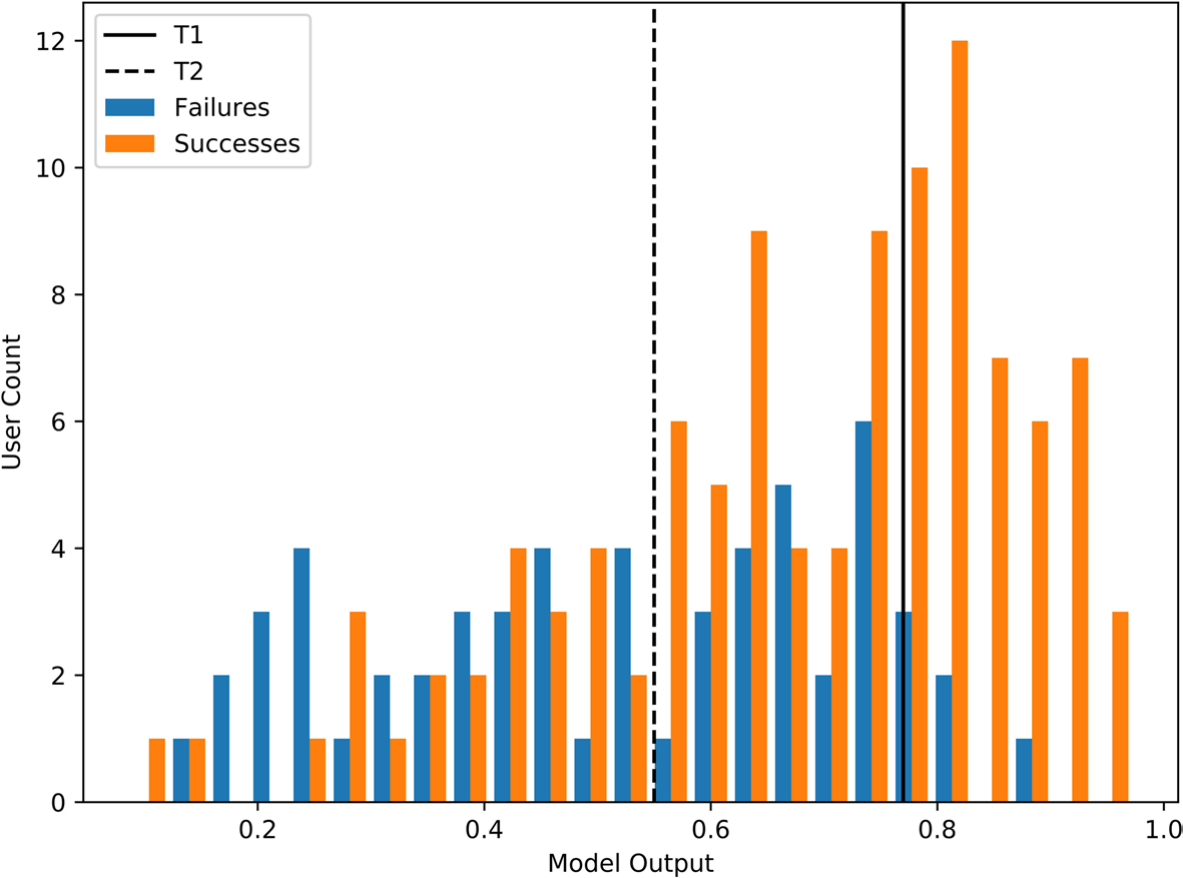
Histogram failure prediction output

1. *What is the purpose and context of the algorithm?* The pain points the respective algorithms address are (1) high dropout rates and (2) low response rates in DMHIs in light of limited resources. The proposed models provide insights into who will likely drop out or not benefit after two out of eight sessions. As such, these predictions serve to adapt individual treatment plans (e.g., through additional guidance, sessions, or reminders) only if and where necessary.
2. *How good were the data used to train the algorithm?* We use the five categories (i.e., completeness, correctness, concordance, plausibility, and currency) to assess data quality for clinical research proposed by the review of Weiskopf and Weng [90]. Regarding completeness, the data consists of all information else provided to the interventions’ e-coach for decision-making. Furthermore, it spans a large variety of what previous work found relevant for intervention dropout and outcome. While additional outside information, such as previous health records or expert assessments, could possibly improve the predictions, the effort necessary to collect them requires extensive steps, deteriorating the cost-value ratio. Since the data stems from RCTs, research staff monitored the completeness of entries and missing data was very low, as seen in supplementary material 1. As for correctness, all non-text dimensions were manually investigated by the two first authors to find mistakes, and data quality was found to be high. The fact that spelling-mistake correction did not increase cross-validation scores indicates a good quality of the text data. Concordance of the data was, for example, internally validated by cross-checking modules completed with the submitted answers, running pivot tables for related variables (e.g., current employment status and leadership responsibility), and ensuring the correct time sequence of the entries. To check for plausibility, every feature’s range and distribution were manually checked by two authors. Questions and findings, including averages and ranges, were discussed with the third author, who was involved in the data collection to check for plausibility, and no issues remained open. The currency of the data is high as the nature of the online setup allows the instant use of the data as soon as the patient submits their answers. As such, a deployed model could inform clinical decisions immediately.
3. *Were there sufficient data to train the algorithm?* The data set at hand is comparatively small for Data Science applications in general, thus presenting one of the major limitations of this study. Especially deep neural networks usually require large amounts of data to perform well. At the same time, this is a prerequisite that is rarely met in E-Mental Health research [52], and with almost 850 participants, the data set is large for DMHI standards. As seen in the related work section, only one other paper considering intervention data exceeds the dataset size presented in this work. A literature review found a dataset size of 100 to be minimally adequate for outcome predictions in DMHIs [91], but only 44% of the 56 studies investigated complied with this criterium. Further, they found that only 29% used a hold-out test set or adequate cross-validation method. At a test set size of 163/170 that was not used for training at any point, the results at hand can be considered among the more generalizable of the works currently available [91]. To address the small dataset size, we extend the pre-training corpus with texts generated by control group users and train the word embeddings at the sentence level. Further, our use of a pre-trained BERT model comes with the significant advantage that - as researchers from a field struggling with data collection - we can leverage large unrelated but available data sets [19]. The results for the deep learning models are stable and good within and across different settings. This suggests an at least minimally adequate data set size for them to compete with classical machine learning models.
4. *How well does the algorithm perform?* With almost all average AUC scores well above 0.5, it can be concluded that the considered features have predictive power regarding intervention outcome and dropout. With the best scores reaching an AUC of 0.70 (dropout) and 0.72 (intervention outcome) after just two weeks, results are competitive with related work. For example, Bremer et al., [18] achieved an AUC of 0.6 when using the user journey data (e.g., time spent) of their first two out of seven sessions to predict dropout. The best prediction models proposed by us achieve a balanced accuracy of 0.66 and 0.67. Forsell et al. [16] did not reach similar balanced accuracy scores predicting outcome with only symptom data until week 3 or 4. The comparison to other related works is limited due to differences in baselines and time horizons. The performance in the sense of clinical usefulness will be discussed in question 8.
5. *Is the algorithm transferable to new clinical settings?* The specific models with their respective (hyper)parameters and, in the case of the NNs, task-specific architecture, can likely not be deployed on a different intervention. However, the proposed process to train the two best-performing models can be replicated on any dataset including intervention text and socio-demographic data. As can be seen in the related work section, these are very common data types to be collected in a standard DMHI setting. The text pre-processing steps are generalizable for any German text and would only have to be slightly adapted for English text (i.e., different handling of capital cases). Transferring models from one language to another in the clinical context has been shown to be possible in other tasks, especially for languages from the same family [92]. The fact that pre-trained neural networks for English text are more in number and more specific in problem-fit [93, 94] indicates that the prediction results of the neural networks could even improve for the English language. Once text features are produced, they can easily serve a variety of outcome measures. The related work section shows several options, ranging from 24 h symptom prediction to 6-month follow-ups. Other options could be to use it to personalize content or adapt the time of intervention.
6. *Are the outputs of the algorithm clinically intelligible?* Considering the transparency of the decision process, neural networks’ black-box nature is one of their major drawbacks. For the non-sequential models, SHAP values and built-in feature importance measures give first insights into the decision-making process. These efforts can easily be extended per the suggestions made by Yang [95] but are left for future research as interpretability is not the focus of this paper. However, the actual outputs of both models are binary and easily understandable as they represent dropout vs. completers and intervention successes vs. failures per the above-given definitions.
7. *How will this algorithm fit into and complement current workflows?* As of now, e-coaches receive general guidelines on how much time to spend with their allocated participants. Within a given RCT, these suggestions did not differ across participants. Once implemented, the models’ predictions could prompt individual suggestions. For example, a stop-light system could indicate green (no risk), yellow (moderate risk), or red (high risk of dropout) [19]. With this information, therapists or e-coaches can decide or be instructed as to which participant is most in need of their time. Pedersen et al. [19] report this approach to have been positively received by therapists in their study. Such risk profiles could also prompt automatic reminders, personalized feedback loops to identify the problem, or additional content (e.g., a module regarding motivation or goal setting).
8. *Has use of the algorithm been shown to improve patient care and outcomes?* The next step to evaluating the practical value of the proposed model is the implementation within a live intervention. However, this exceeds the limits of this paper. At the same time, studies such as Forsell et al. [16] and Pedersen et al. [19] have empirically proven the superiority of adaptive care for both dropout and outcome predictions. In the baseline, the limited resources are currently being distributed at random. Empirical evidence shows that many patients benefit from unguided interventions [13] and Forsell et al. [16] show that at-risk patients — while significantly benefiting — even with enhanced care, barely reach the same health outcomes as not at-risk counterparts.
  The best model predicting outcome recognizes 93% of intervention failures (recall) while avoiding overspending on 41% of the most likely completers (specificity). The same calculations for the slightly less balanced dropout predictions lead to 55% correctly identified dropouts while avoiding overspending on 80% of completers. These metrics can be off-traded through the threshold deciding between a dropout or failure, as exemplarily shown in Fig. 2. The histograms show the intervention failure probability as predicted by the winning model for each group – intervention failures and successes. As expected, successes have a higher probability of being recognized as such (right side), failures are more prevalent in the low probabilities (left side), and there is a bulk of hard-to-identify participants in the middle. Changing the threshold from T1 (highest balance accuracy) to T2 decreases the recall to 53%; however, it avoids overspending on 75% of successes. Consequently, not much more than one-third of all participants receive enhanced care, lowering costs significantly while still addressing those most likely participants to benefit from support. The threshold can be adapted to fit the available resources and can even inform the number of participants accepted in the intervention. Considering the preventive nature of the stress intervention at hand, one application of the model could be to make the intervention available without guidance to reach as many participants as possible and only offer the available guidance to those who most need it. In the T2 scenario in Fig. 2, this increases the number of participants reached by threefold.
9. *Could the algorithm cause patient harm?* The purpose is to optimize resource allocation while maintaining or improving the level of care over the entire population of participants. If it were used to reduce the average care level, it could harm those incorrectly classified as completers or successes through decreased levels of care. Such as prospect is especially worrisome when working with a population with severe symptoms. Depending on the importance of avoiding such false negatives, the recall can be increased at higher costs of resources. It, thus, must always be closely considered *how* to implement such a decision-support tool in *which* setting. At the same time, considering that right now, very limited resources are available, and many sick people are not being helped at all, increasing the total number of participants treated is a factor to weigh in with individual effects.
10.  *Does use of the algorithm raise ethical, legal or social concerns?* Albeit the focus of early research being on establishing the overall feasibility, bias in the data must be considered early on. With primarily female participants that hold a university degree, the data at hand is — while typical for mental health interventions — not representative of the general population. Implementing such a model in routine care could disadvantage those groups with the already most extensive unmet needs and must be adjusted to ensure the best possible care for all [96]. In addition, ethical and legal aspects of an automated decision to change the level of care must be closely considered, especially in cases where the reason for the prediction is not transparent [95].

## Conclusion

NLP methods can help make countless individual predictions based on text that would require impossible amounts of human resources to be analyzed. While the use of sophisticated NLP methods on non-clinical texts is continuously advancing in Mental Health diagnostics [30, 31, 35, 50], applications of NLP on E-mental health intervention text have been few and predominantly limited to simple models. In this case study, we train several ML models, considering various text representation methods and additional data inputs, to predict intervention failure and intervention dropout. For this, we use a dataset of 849 German-speaking participants of a stress intervention. By thoroughly evaluating combinations of the above-mentioned factors on our dataset, we contribute to the design choice of prediction models for intervention dropout and intervention outcome.

First, we demonstrate that *harnessing the sequential nature of text* by training deep learning models in combination with word embeddings outperforms the much simpler approach of using averaged word vectors on our test set. Thus, we complement existing research [25, 27] by proposing a task-specific LSTM architecture using word embeddings which successfully deals with the long input sequences and yields good results (average AUC score of 0.65) in dropout prediction. We further demonstrate the need to treat the embedding dimension as a hyperparameter rather than using the default values. Second, considering *supplementary baseline* *data* when predicting intervention outcome and *evaluation data* when predicting dropout yields the best-performing models. Thus, our findings support that the participants’ background and attitude towards the intervention hold additional information in combination with text data. Third, we underline the *solid performance of easy-to-implement approaches* to predict dropout (simple meta-data and TF-IDF) and intervention outcome (advanced meta-data and sentiment analysis). By providing the insights from our case study, we seek to facilitate the development of ML-based tools which augment e-coaches’ work in extracting valuable information from the participants’ intervention texts — hence, easing the task of identifying participants in need of human attention. With these predictions, necessary steps towards a more successful intervention in light of limited resources to face growing needs can be initiated.

Considering the still relatively small data set size and high specificity of our intervention set-up, this research is only a step towards better understanding, predicting, and ultimately influencing participants' behavior in DMHIs. Data sets such as this one can be considered the most promising approach to gathering knowledge in this research area. Yet, learning on few data points might not champion the same text representation methods and models, and more research is necessary to determine the generalizability of our findings. While we prove the potential of neural networks in this setting, they require large datasets, long training times, and have a black-box nature. However, the investigation of such complex methods is necessary to ensure the best possible results — especially considering the astonishing results deep learning models achieve on other NLP tasks. To truly understand human language, words must be considered beyond their lexical meaning, and the specific context needs to be understood — a task simple methods will never solve. One further way to address the problem of small datasets could be to use data augmentation methods as commonly used in computer vision, and more recently proposed for NLP tasks [97]. We suggest that employing attention-based [98] deep learning architectures can further enhance the model performance in prediction tasks such as ours. While designing task-specific network architectures like ours may be a complex and tedious task, large pre-trained text classification models can eliminate this work. To determine whether further research in applying pre-trained transformer models in this domain is auspicious, we examine the most prominent transformer model BERT and observe promising results in dropout prediction. Thus, we suggest investigating more sophisticated pre-trained transformer models (e.g., RoBERTa [99] or XLNet [100]) in such settings. In addition to an optimized pre-training strategy, XLNet tends to process long sentences better than BERT, which could be advantageous in cases like ours and further improve the model performance. Besides the particular transformer model, the text corpora used for pre-training, as well as the approaches to integrating the important non-text features into the model architecture, should be investigated in more detail (e.g., [101]). Furthermore, multi-task models (e.g., predicting intervention failure and dropout at the same time), which are frequently employed in other NLP tasks (e.g., [102]), can potentially improve results on both tasks. For the time being, simple feature representations such as metadata and classical statistical models should be considered an easy-to-implement yet competitive option for predicting intervention failure and dropout. In that regard, further research must be conducted to investigate how to improve these predictions, for example, more automized ways of finding the most important TF-IDF features [103].

### Supplementary Information

Below is the link to the electronic supplementary material.

Supplementary file1 (PDF 91 KB)

Supplementary file2 (PDF 154 KB)

## Data Availability

Due to the sensitivity of text data in the context of DMHIs, the data cannot be made available. The source code can be made available upon request to the corresponding author.

## References

[1] Wang PS, Lane M, Olfson M, Pincus HA, Wells KB, Kessler RC (2005) Twelve-month use of mental health services in the United States. JAMA Psychiatry 62(6):629–640. 10.1001/archpsyc.62.6.629 10.1001/archpsyc.62.6.629 15939840

[2] Rommel A, Bretschneider J, Kroll LE, Prütz F, Thom J (2017) Inanspruchnahme psychiatrischer und psychotherapeutischer Leistungen – Individuelle Determinanten und regionale Unterschiede. J Health Monit 68(08):e31

[3] Santomauro DF, Herrera AMM, Shadid J, Zheng P, Ashbaugh C, Pigott DM, Abbafati C, Adolph C, Amlag JO, C.-1. M. D. Collaborators (2021) Global prevalence and burden of depressive and anxiety disorders in 204 countries and territories in 2020 due to the COVID-19 pandemic. Lancet 398(10312):1700–1712. 10.1016/S0140-6736(21)02143-7 10.1016/S0140-6736(21)02143-7 PMC8500697 34634250

[4] Ebert DD, Harrer M, Apolinário-Hagen J, Baumeister H (2019) Digital interventions for mental disorders: key features, efficacy, and potential for artificial intelligence applications, In Frontiers in Psychiatry, Singapore, Springer Natur, pp 584–627 10.1007/978-981-32-9721-0_29 31705515

[5] Karyotaki E, Kleiboer A, Smit F, Turner D, Pastor A, Andersson G, Berger T, Botella C, Breton J, Carlbring P, Christensen H, de Graaf E, Griffiths K, Donker T, Farrer L, Huibers M, Lenndin J, Mackinnon A, Meyer B, Moritz S, Riper R (2015) Predictors of treatment dropout in self-guided web-based interventions for depression: an ‘individual patient data’ meta-analysis. Psychol Med 45(13):2717–2726. 10.1017/S0033291715000665 10.1017/S0033291715000665 25881626

[6] Andersson G, e Carlbring, Rozental A (2019) Response and remission rates in internet-based cognitive behavior therapy: an individual patient data meta-analysis. Front Psychiatry 10. 10.3389/fpsyt.2019.00749 10.3389/fpsyt.2019.00749 PMC6823683 31708813

[7] Heber E, Ebert DD, Lehr D, Cuijpers P, Berking M, Nobis S, Riper H (2017) The benefit of web- and computer-based interventions for stress: a systematic review and meta-analysis. J Med Internet Res 19(2):e32. 10.2196/jmir.5774 10.2196/jmir.5774 PMC5336602 28213341

[8] Reins JA, Buntrock C, Zimmermann J, Grund S, Harrer M, Lehr D, Baumeister H, Weisel K, Domhardt M, Imamura K, Kawakami N, Spek V, Nobis S, Snoek F, Cuijpers P, Klein JP, Moritz S (2021) Efficacy and moderators of internet-based interventions in adults with subthreshold depression: an individual participant data meta-analysis of randomized controlled trials. Psychother Psychosom 90(2):94–106. 10.1159/000507819 10.1159/000507819 32544912

[9] Karyotaki E, Ebert DD, Donkin L, Riper H, Twisk J, Burger S, Rozental A, Lange A, Williams AD, Zarski AC, Geraedts A, Straten Av, Kleiboer A, Meyer B, Ince BBÜ, Buntro C (2018) Do guided internet-based interventions result in clinically relevant changes for patients with depression? An individual participant data meta-analysis. Clin Psychol Rev 63:80–92. 10.1016/j.cpr.2018.06.007 10.1016/j.cpr.2018.06.007 29940401

[10] Domhardt M, Letsch J, Kybelka J, Koenigbauer J, Doebler P, Baumeister H (2020) Are Internet- and mobile-based interventions effective in adults with diagnosed panic disorder and/or agoraphobia? A systematic review and meta-analysis. J Affected Disord 276:169–182. 10.1016/j.jad.2020.06.059 10.1016/j.jad.2020.06.059 32697696

[11] Donkin L, Christensen H, Naismith SL, Neal B, Hickie IB, Glozier N (2011) A systematic review of the impact of adherence on the effectiveness of e-therapies. J Med Internet Res 13(3):e52. 10.2196/jmir.1772 10.2196/jmir.1772 PMC3222162 21821503

[12] Gan DZQ, McGillivray L, Han J, Christensen H, Torok M (2021) Effect of engagement with digital interventions on mental health outcomes: a systematic review and meta-analysis. Front Digit Health 3. 10.3389/fdgth.2021.764079 10.3389/fdgth.2021.764079 PMC8599127 34806079

[13] Richards D, Richardson T (2012) Computer-based psychological treatments for depression: a systematic review and meta-analysis. Clin Psychol Rev 10.1016/j.cpr.2012.02.004 22466510

[14] Baumeister H, Reichler L, Munzinger M, Lin J (2014) The impact of guidance on Internet-based mental health interventions — a systematic review. Internet Interv 1(4):205–215. 10.1016/j.invent.2014.08.003

[15] Hilvert-Bruce Z, Rossouw PJ, Wong N, Sunderland M, Andrews G (2012) Adherence as a determinant of effectiveness of internet cognitive behavioural therapy for anxiety and depressive disorders. Behav Res Ther 50(7-8):463–468. 10.1016/j.brat.2012.04.001 10.1016/j.brat.2012.04.001 22659155

[16] Forsell E, Jernelöv S, Blom K, Kraepelien M, Svanborg, Andersson G, Lindefors N, Kaldo V (2019) Proof of concept for an adaptive treatment strategy to prevent failures in internet-delivered CBT: a single-blind randomized clinical trial with insomnia patient. Am J Psychiatry 176(4):315–323. 10.1176/appi.ajp.2018.18060699 10.1176/appi.ajp.2018.18060699 30696270

[17] Shatte ABR, Hutchinson DM, Teague SJ (2019) Machine learning in mental health: a systematic scoping review of methods and applications. Psychol Med 49(9):1426–1448 10.1017/S0033291719000151 30744717

[18] Bremer V, Chow PI, Funk B, Thorndike FP, Ritterband LM (2020) Developing a process for the analysis of user journeys and the prediction of dropout in digital health interventions: machine learning approach. J Med Internet Res 22(10) 10.2196/17738 PMC7657718 33112241

[19] Pedersen DH, Mansourvar M, Sortsø C, Schmidt T (2019) Predicting dropouts from an electronic health platform for lifestyle interventions: analysis of methods and predictors. J Med Internet Res 21(9). 10.2196/13617 10.2196/13617 PMC6753691 31486409

[20] Chekroud A, Bondar J, Delgadillo J, Doherty G, Wasil A, Fokkema M, Cohen Z, Belgrave D, DeRubeis R, Iniesta R, Dwyer D, Choi K (2021) The promise of machine learning in predicting treatment outcomes in psychiatry. World Psychiatry 20(2):154–170. 10.1002/wps.20882 10.1002/wps.20882 PMC8129866 34002503

[21] Corcoran CM, Benavides C, Cecchi G (2019) Natural language processing: opportunities and challenges for patients, providers, and hospital systems. Psychiatr Annu 49(5):202–208. 10.3928/00485713-20190411-01

[22] Abbe A, Grouin C, Zweigenbaum P, Falissard B (2015) Text mining applications in psychiatry: a systematic literature review. Int J Methods Psychiatr Res 25(2):86–100. 10.1002/mpr.1481 10.1002/mpr.1481 PMC6877250 26184780

[23] Calvo R, Milne D, Hussain M, Christensen H (2017) Natural language processing in mental health applications using non-clinical texts. Nat Lang Eng 23(5):649–685. 10.1017/S1351324916000383

[24] Bone D, Lee C-C, Chaspari T, Gibson J, Narayanan S (2017) Signal processing and machine learning for mental health research and clinical applications. IEEE Signal Process Magazin 34(5):196–195. 10.1109/MSP.2017.2718581

[25] Funk B, Sadeh-Sharvit S, Fitzsimmons-Craft E, Trockel M, Monterubio G, Goel N, Balantekin K, Eichen D, Flatt R, Firebaugh M-L, Jacobi C, Graham A, Hoogendoorn M (2020) A framework for applying natural language processing in digital health interventions. J Med Internet Res 22(2):e13855. 10.2196/13855 10.2196/13855 PMC7059510 32130118

[26] Hoogendoorn M, Berger T, Schulz A, Stolz T, Szolovits P (2016) Predicting social anxiety treatment outcome based on therapeutic email conversations. IEEE J Biomed Health Inform 21(5):1449–1459. 10.1109/JBHI.2016.2601123 10.1109/JBHI.2016.2601123 PMC5613669 27542187

[27] Gogoulou E, Boman M, Abdesslem FB, Isacsson N, Kaldo V, Sahlgren M (2021) Predicting treatment outcome from patient texts: the case of internet-based cognitive behavioural therapy. In: Proceedings of the 16th Conference of the European Chapter of the Association for Computational Linguistics pp 575–580. 10.18653/v1/2021.eacl-main.46

[28] Bengio Y, Ducharme R, Vincent P, Jauvin C (2003) A neural probabilistic language model. J Mach Learn Res 3:1137–1155

[29] Devlin J, Chang M-W, Lee K, Toutanova K (2019) BERT: pre-training of deep bidirectional transformers for language understanding. In: Proceedings of the 2019 Conference of the North American Chapter of the Association for Computational Linguistics: Human Language Technologies, Minneapolis, Minnesota 1:4171–4186. 10.18653/v1/N19-1423

[30] Nobles AL, Glenn JJ, Kowsari K, Teachman eA, Barnes LE (2018) Identification of imminent suicide risk among young adults using text messages. In: Nobles AL et al (ed) Identification of Imminent Suicide Risk Among Young Adults using Text Messages. Proceedings of the SIGCHI conference on human factors in computing systems. CHI Conference, pp 1–11. 10.1145/3173574.3173987 10.1145/3173574.3173987 PMC6442737 30944915

[31] Cohan A, Desmet B, Yates A, Soldaini L, MacAvaney S, Goharian N (2018) SMHD: a large-scale resource for exploring online language usage for multiple mental health conditions. In: Proceedings of the 27th International Conference on Computational Linguistics, Santa Fe pp 1485–1497

[32] Howes C, Purver M, McCabe R (2014) Linguistic indicators of severity and progress in online text-based therapy for depression. Association for Computational Linguistics. In: Workshop on Computational Linguistics and Clinical Psychology: From Linguistic Signal to Clinical Reality, Baltimore pp 7–16. 10.3115/v1/W14-3202

[33] Calvo R, Milne DN, Hussain S, Christensen H (2017) Natural language processing in mental health applications using non-clinical texts 23(5):649–685

[34] Oesterreich TD, Fitte C, Behne A, Teuteberg F (2020) Understanding the role of predictive and prescriptive analytics in healthcare: a multi-stakeholder approach. In: Proceedings of the 28th European Conference on Information Systems (ECIS) 28:1–19

[35] Wołk A, Chlasta K, Holas P (2021) Hybrid approach to detecting symptoms of depression in social media entries, in Pacific Asia Conference on Information Systems Proceedings, Dubai, UAE

[36] Tsang EW (2014). Case studies and generalization in information systems research: a critical realist perspective. J Strat Inf Syst.

[37] Eloranta S, Boman M (2022) Predictive models for clinical decision making: deep dives in practical machine learning. J Intern Med 292(2):278–295. 10.1111/joim.13483 10.1111/joim.13483 PMC9544754 35426190

[38] Cepoiu M, McCusker J, Cole MG, Sewitch M, Belzile E, Ciampi A (2007) Recognition of depression by non-psychiatric physicians—a systematic literature review and meta-analysis. J Gen Intern Med 23(1):25–36. 10.1007/s11606-007-0428-5 10.1007/s11606-007-0428-5 PMC2173927 17968628

[39] DeMasi O, Kording K, Recht B (2017) Meaningless comparisons lead to false optimism in medical machine learning. PLoS One 12(9):e0184604. 10.1371/journal.pone.0184604 10.1371/journal.pone.0184604 PMC5614525 28949964

[40] Becker D, Breda Wv, Funk B, Hoogendoorna M, Ruwaardc J, Riperc H (2018) Predictive modeling in e-mental health: a common language framework. Internet Interv 12:57–67. 10.1016/j.invent.2018.03.002 10.1016/j.invent.2018.03.002 PMC6096321 30135769

[41] Le Glaz A, Haralambous Y, Kim-Dufor D-H, Lenca P, Billot R, Ryan TC, Marsh J, DeVylder J, Walter M, Berrouiguet S, Lemey C (2021) Machine learning and natural language processing in mental health: systematic review. J Med Internet Res 23(5):e15708. 10.2196/15708 10.2196/15708 PMC8132982 33944788

[42] Paul A, Liao W-k, Alok Choudhary AA (2021). Harnessing psycho-lingual and crowd-sourced dictionaries for predicting taboos in written emotional disclosure in anonymous confession boards. J Healthc Inform Res.

[43] Masino AJ, Forsyth D, Fiks AG (2018). Detecting adverse drug reactions on twitter with convolutional neural networks and word embedding features. J Health Inform Res.

[44] Yeruva VK, Junaid S, Lee Y (2019). Contextual word embeddings and topic modeling in healthy dieting and obesity. J Healthc Inform Res.

[45] Spärck Jones K (1972) A statistical interpretation of term specificity and its application in retrieval. J Doc 28(1):11–21. 10.1108/eb026526

[46] Marcus MD, Wildes JE (2012) Obesity in DSM-5. Psychiatr Ann 42(11):431–435. 10.3928/00485713-20121105-10

[47] Wongkoblap A, Vadillo M, Curcin V (2021) Depression detection of twitter posters using deep learning with anaphora resolution: algorithm development and validation. J Med Internet Res Ment Health 8(8). 10.3390/electronics11050676 10.2196/19824 PMC8380581 34383688

[48] Pennebaker J, Boyd R, Jordan K, Blackburn K (2015). The development and psychometric properties of LIWC2015.

[49] Coppersmith G, Carvalho P, Silva MJ, Wallace BC, Amir S (2017) Quantifying mental health from social media with neural user embeddings. In: Proceedings of the 2nd Machine Learning for Healthcare Conference, Boston 68:306–321

[50] Bucur A-M, Cosma A, Dinu LP (2021) Early risk detection of pathological gambling, self-harm and depression using BERT. In: Proceedings of Conference and Labs of the Evaluation Forum, Bucharest, Romania

[51] Ewbank MP, Cummins R, Tablan V, Bateup S, Catarino A, Martin AJ, Blackwell AD (2020) Quantifying the association between psychotherapy content and clinical outcomes using deep learning. JAMA Psychiatry 77(1):35–43. 10.1001/jamapsychiatry.2019.2664 10.1001/jamapsychiatry.2019.2664 PMC6707006 31436785

[52] Pasini A (2015) Artificial neural networks for small dataset analysis. J Thorac Dis 7(5). 10.3978/j.issn.2072-1439.2015.04.61 10.3978/j.issn.2072-1439.2015.04.61 PMC4454870 26101654

[53] Eysenbach G (2005) The law of attrition. J Med Internet Res 7(1):1–9. 10.2196/jmir.7.1.e11 10.2196/jmir.7.1.e11 PMC1550631 15829473

[54] Pihlaja S, Lahti J, Lipsanen JO, Ritola V, Gummerus E-t, Stenberg J-H, Joffe G (2020) Scheduled telephone support for internet cognitive behavioral therapy for depression in patients at risk for dropout: pragmatic randomized controlled trial. J Med Internet Res 22(7):e15732. 10.2196/15732 10.2196/15732 PMC7413288 32706658

[55] Smink WAC, Sools AM, Postel MG, Sang ETK, Elfrink A, Libbertz-Mohr LB, Veldkamp BP, Westerhof GJ (2021) Analysis of the emails from the Dutch web-based intervention “Alcohol de Baas”: assessment of early indications of drop-out in an online alcohol abuse intervention. Front Psychiatry 12:575931. 10.3389/fpsyt.2021.575931 10.3389/fpsyt.2021.575931 PMC8714780 34975551

[56] Grave E, Joulin A, Mikolov T, Bojanowski P (2017). Enriching word vectors with subword information. Trans Assoc Comput Linguist.

[57] Le Q, Mikolov T (2014) Distributed representations of sentences and documents. In: In International conference on machine learning, Beijing 32(2):1188–1196

[58] Mikolov T, Grave E, Bojanowski P, Puhrsch C, Joulin A (2017) Advances in pre-training distributed word representations. arXiv:1712.09405

[59] Blumer A, Ehrenfeucht A, Haussler D, Warmuth MK (1987) Occam’s Razor. Inf Process Lett 24(6):377–380. 10.1016/0020-0190(87)90114-1

[60] D'Zurilla TJ, Nezu AM (2010) Problem-solving therapies. In: Handbook of cognitive–behavioral therapies, vol 3. Guilford Press, pp 197–225

[61] Berking M, Whitley B (2014). Affect regulation training - a practitioners’ manual, New York.

[62] Heber E, Lehr D, Ebert DD, Berking M, Riper H (2016) Web-based and mobile stress management intervention for employees: a randomized controlled trial. J Med Internet Res 18(1) 10.2196/jmir.5112 PMC4749847 26818683

[63] Ebert DD, Lehr D, Heber E, Riper H, Cuijpers P, Berking M (2016). Internet- and mobile-based stress management for employees with adherence-focused guidance: efficacy and mechanism of change. Scand J Work Environ Health.

[64] Ebert DD, Heber E, Berking M, Riper H, Cuijpers P, Funk B, Lehr D (2016). Self-guided internet-based and mobile-based stress management for employees: results of a randomised controlled trial. Occup Environ Med.

[65] Nixon P, Ebert DD, Boß L, Angerer P, Dragano N, Lehr D (n.d.) Web-based stress management intervention for employees experiencing effort-reward imbalance at work: a randomized controlled trial. Preprint

[66] Ebert DD, Franke M, Zarski A-C, Berking M, Riper H, Cuijpers P, Funk B, Lehr D (2021) Effectiveness and moderators of an internet-based mobile-supported stress management intervention as a universal prevention approach: randomized controlled trial. J Med Internet Res 23(12):e22107. 10.2196/22107 10.2196/22107 PMC8734929 34941541

[67] Nixon P, Ebert DD, Boß L, Angerer P, Dragano N, Lehr D (2022) Efficacy of a web-based stress management intervention for employees experiencing adverse working conditions and occupational self-efficacy as mediator: a randomized controlled trial. J Med Internet Res 24(10). 10.2196/40488 10.2196/40488 PMC9634524 36264607

[68] Cohen S, Kamarck T, Mermelstein R (1983) A global measure of perceived stress. J Health Soc Behav 24(4):385–396. 10.2307/2136404 6668417

[69] Schneider EE, Schönfelder S, Domke-Wolf M, Wessa M (2020) Measuring stress in clinical and nonclinical subjectsusing a German adaptation of the Perceived StressScale. Int J Clin Health Psychol 10.1016/j.ijchp.2020.03.004 PMC7296237 32550857

[70] Jacobson NS, Truax P (1991) Clinical significance: a statistical approach to defining meaningful change in psychotherapy research. J Consult Clin Psychol 59(1):12–19. 10.1037/0022-006X.59.1.12 10.1037//0022-006x.59.1.12 2002127

[71] Christensen H, Griffithi KM, Farrer L (2009) Adherence in internet interventions for anxiety and depression: systematic review. J Med Internet Res 11(2):e13. 10.2196/jmir.1194 10.2196/jmir.1194 PMC2762797 19403466

[72] Hedman E, Ljótsson B, Kaldo V, Hesser H, Alaoui SE, Kraepelien M, Andersson E, Rück C, Svanborg C, Andersson G, Lindefors N (2014) Effectiveness of Internet-based cognitive behaviour therapy for depression in routine psychiatric care. J Affect Disord 155:49–58. 10.1016/j.jad.2013.10.023 10.1016/j.jad.2013.10.023 24238951

[73] Cook BL, Progovac AM, Chen P, Mullin B, Hou S, Baca-Garcia E (2016) Novel use of natural language processing (NLP) to predict suicidal ideation and psychiatric symptoms in a text-based mental health intervention in Madrid. Comput Math Methods Med 2016:8708434. 10.1155/2016/8708434 10.1155/2016/8708434 PMC5056245 27752278

[74] Fehle J, Schmidt T, Wolff C (2021) Lexicon-based sentiment analysis in German: systematic evaluation of resources and preprocessing techniques. In: Proceedings of the 17th Conference on Natural Language Processing, Düsseldorf pp 86–103

[75] Camacho-Collados J, Pilehvar MT (2018) On the role of text preprocessing in neural network architectures: an evaluation study on text categorization and sentiment analysis. In: Proceedings of the 2018 Conference of Empirical Methods in Natural Language Processing Workshop BlackboxNLP: Analyzing and Interpreting Neural Networks for NLP, Brussels

[76] Blei DM, Ng AY, Jordan MI (2003) Latent Dirichlet allocation. J Mach Learn Res 3:993–1022

[77] Chawla NV, Bowyer KW, Hall LO, Kegelmeyer WP (2002) SMOTE: synthetic minority over-sampling technique. J Artif Intell Res 16(1):321–357 10.1613/jair.953

[78] Cortes C, Vapnik V (1995) Support-vector networks. Mach Learn

[79] Chen T, Guestrin C (2016) XGBoost: a scalable tree boosting system, In Knowledge Discovery and Data Mining, San Francisco

[80] Guyon I, Saffari A, Dror G, Cawley G (2011) Model selection: beyond the Bayesian/Frequentist divide. J Mach Learn Res 61–87

[81] Schapire RE (2013) Explaining AdaBoost, In Empirical Inference, Heidelberg, Springer-Verlag Berlin Heidelberg

[82] Schuster M, Paliwal KK (1997) Bidirectional recurrent neural networks. IEEE Trans Sig Process 2673–2681. 10.1109/78.650093

[83] Hochreiter Sepp, Schmidhuber Jürgen (1997). Long short-term memory. Neural Comput.

[84] Bradley AP (1997) The use of the area under the ROC curve in the evaluation of machine learning algorithms. Pattern Recognit 30(7):1145–1159. 10.1016/S0031-3203(96)00142-2

[85] Olczak J, Pavlopoulos J, Prijs J, Ijpma FFA, Doornberg JN, Lundström C, Hedlund J, Gordon M (2021). Presenting artificial intelligence, deep learning, and machine learning studies to clinicians and healthcare stakeholders: an introductory reference with a guideline and a Clinical AI Research (CAIR) checklist proposal. Acta Orthop.

[86] Lundberg SM, Lee S-I (2017) A unified approach to interpreting model predictions. In: Conference on Neural Information Processing Systems, Long Beach pp 4768–4777

[87] Barrett MS, Chua W-J, Crits-Christoph P, Gibbons MB, Casiano D, Thompson D (2008) Early withdrawal from mental health treatment: implications for psychotherapy practice. Psychotherapy 45(2):247–267. 10.1037/0033-3204.45.2.247 10.1037/0033-3204.45.2.247 PMC2762228 19838318

[88] Cabitza F, Campagner A (2021) The need to separate the wheat from the chaff in medical informatics. Int J Med Inform 153:104510. 10.1016/j.ijmedinf.2021.104510 10.1016/j.ijmedinf.2021.104510 34108105

[89] Scott I, Carter S, Coiera E (2021) Clinician checklist for assessing suitability of machine learning applications in healthcare. BMJ Health Care Inf 28:e100251. 10.1136/bmjhci-2020-100251 10.1136/bmjhci-2020-100251 PMC7871244 33547086

[90] Weiskopf NG, Wenig C (2013). Methods and dimensions of electronic health record data quality assessment: enabling reuse for clinical research. J Am Med Inform Assoc.

[91] Sajjadian M, Lam RW, Milev R, Rotzinger S, Frey BN, Soares CN, Parikh SV, Foster JA, Turecki G, Müller DJ, Strother SC, Farzan F, Kennedy SH, Uher R (2021). Machine learning in the prediction of depression treatment outcomes: a systematic review and meta-analysis. Psychol Med.

[92] Névéol A, Dalianis H, Velupillai S, Savova G, Zweigenbaum P (2018). clinical natural language processing in languages other than English: opportunities and challenges. J Biomed Semant.

[93] Ji S, Zhang T, Ansari L, Fu J, Tiwari P, Cambria E (2021) MentalBERT: publicly available pretrained language models for mental healthcare. Comput Lang. 10.48550/arXiv.2110.15621

[94] Hugging Face, huggingface model overview, [Online]. Available: https://huggingface.co/models?language=de&sort=downloads. Accessed 23 09 2022

[95] Yang CC (2022). Explainable artificial intelligence for predictive modeling in healthcare. J Healthc Inform Res.

[96] Gianfrancesco M, Tamang S, Yazdany J, Schmajuk G (2018) Potential biases in machine learning algorithms using electronic health record data. JAMA Intern Med 178(11):1544–1547. 10.1001/jamainternmed.2018.3763 10.1001/jamainternmed.2018.3763 PMC6347576 30128552

[97] Xiang R, Chersoni E, Lu Q, Huang CR, Li W, Long Y (2021). Lexical data augmentation for sentiment analysis. J Am Soc Inf Sci.

[98] Vaswani A, Shazeer N, Parmar N, Uszkoreit J, Jones L, Gomez A, Kaiser L, Polosukhin (2017) Attention is all you need. In: Proceedings of the 31st International Conference on Neural Information Processing Systems (NIPS). NIPS, Long Beach, CA, USA, pp 6000–6010

[99] Yang Z, Dai Z, Yang Y, Carbonell J, Salakhutdinov R, Le QV (2019) XLNet: generalized autoregressive pretraining for language understanding, Proceedings of the 33rd International Conference on Neural Information Processing Systems., Curran Associates Inc., Red Hook, 517:5753–5763

[100] Liu Y, Ott M, Goyal N, Du J, Joshi M, Chen D, Levy O, Lewis M, Zettlemoyer L, Stoyanov V (2019) RoBERTa: a robustly optimized BERT pretraining approach. arxiv 1907.11692

[101] Shen JX, Ma MD, Xiang R, Lu Q, Vallejos EP, Xu G, Huang CR, Long Y (2020). Dual memory network model for sentiment analysis of review text. Knowl-Based Syst.

[102] Hashimoto K, Xiong C, Tsuruoka Y, Socher R (2017) A joint many-task model: growing a neural network for multiple NLP tasks. In: Proceedings of the 2017 Conference on Empirical Methods in Natural Language Processing, Copenhagen, Denmark, 1923–1933. 10.18653/v1/D17-1206

[103] Zhang Y, Zhou Y, Yao J (2020) Feature extraction with TF-IDF and game-theoretic shadowed sets communications in computer and information science. In: Information Processing and Management of Uncertainty in Knowledge-Based Systems, vol 1237. Springer, Cham, pp 722–733. 10.1007/978-3-030-50146-4_53

