# Supplementary material for: Finding the Best Match — a Case Study on the (Text-)Feature and Model Choice in Digital Mental Health Interventions
Source: J Healthc Inform Res. 2023 Sep 18;7(4):447–79. doi: 10.1007/s41666-023-00148-z (PMC10620349; doi:10.1007/s41666-023-00148-z)
Supplement: Supplementary file 1 — Supplementary file1 (PDF 91 KB) [file 41666_2023_148_MOESM1_ESM.pdf]

"Finding the Best Match – A Case Study on the (Text-)Feature and Model Choice in Digital Mental Health Interventions"

Zantvoort, Scharfenberger, Boß, Lehr, Funk; Leuphana University (kirsten.zantvoort@leuphana.de)

Journal of Health Informatic Research - Special Issue: Health Natural Language Processing

**Data Types**	
C = Continous	
D = Dummy Variables	
O = Ordinal Encoding	

**Sample Size**		
n	849	
**Age, mean (SD)**		C
Age in years	42.13 (9.68)	
**Gender**		D
Female	659	
Male	189	
Other	1	
**Family Status**		D
Single	352	
Partnership	497	
**Children**		D
No Children	437	
Children in same household	300	
Childre in other household	112	
**Highschool Education**		O
Highest	619	
Middle	213	
Lowest	16	
Special Needs School	1	
**Highest Education**		O
University	512	
Community college	130	
Vocational education	196	
None	11	

**Occupation**		D
Full-time	647	
Part-time	190	
None	12	
**Leadership Responsibility**		D
Yes	333	
No	516	
**Working Contract**		D
Permanent	612	
Temporary	93	
State official	74	
Self-employed	58	
Other	12	
**Income (€ p.a.)**		O
Not reported	77	
100k	17	
60-100k	90	
50-60k	107	
40-50k	135	
30-40k	193	
10-30k	210	
>10k	20	
**Area of Work**		D
Social sector	182	
Service sector	157	
Health	140	
Business/ administratio	136	
Information technology	61	
Others	173	

Guidance level		O
Self-help	378	
On demand	355	
Guidance	116	
**Dropout**		D
Dropout	197	
Completer	652	
**Intervention Outcome**		D
Success	511	
Failure	298	
Missing	40	
**Symptom Score Sum PSS (range), mean (SD)**		C
Pre-intervention (6-37)	24.9 (4.79)	
Post-intervention (2-37)	17.2 (6.11)	
**Baseline Subscores PSS (range), mean (SD)**		C
Helplessness (2-24)	15.8 (3.41)	
Self Efficiacy (0-15)	9.06 (2.14)	
**Previous Experience with Similar Training**		D
Yes	104	
No	745	
**Experience Psychotherapy**		D
Yes, previously.	301	
Yes, currently	70	
No.	478	
**Previous Experience Stress Intervention**		D
Yes	102	
No	747	
**Participant Recruited Via Health Insurance**		D
Yes	570	
No	279	
