# Supplementary material for: Finding the Best Match — a Case Study on the (Text-)Feature and Model Choice in Digital Mental Health Interventions
Source: J Healthc Inform Res. 2023 Sep 18;7(4):447–79. doi: 10.1007/s41666-023-00148-z (PMC10620349; doi:10.1007/s41666-023-00148-z)
Supplement: Supplementary file 2 — Supplementary file2 (PDF 154 KB) [file 41666_2023_148_MOESM2_ESM.pdf]

"Finding the Best Match – A Case Study on the (Text-)Feature and Model Choice in Digital Mental Health Interventions"

Zantvoort, Scharfenberger, Boß, Lehr, Funk; Leuphana University (kirsten.zantvoort@leuphana.de)

Journal of Health Informatic Research - Special Issue: Health Natural Language Processing

Fit im Stress - Lektion 2

Verabreden Sie sich

Damit Ihr gutes Vorhaben in der Hektik des Alltags auch nicht untergeht, kann es helfen, sich einen festen Termin für das Training einzuplanen. Überlegen Sie sich bitte nun hier wieder, *wann* Sie die nächste Lektion bearbeiten möchten, d.h. an welchem Tag und in etwa zu welcher Uhrzeit.

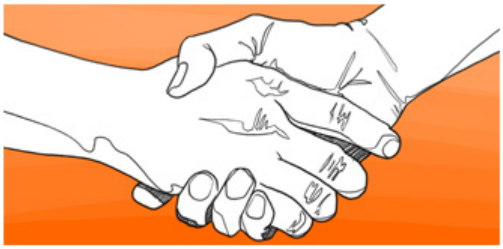

Um optimal von dem Programm profitieren zu können, empfehlen wir Ihnen, mindestens eine Lektion pro Woche zu bearbeiten, **am besten eine bis zwei Lektionen pro Woche**.

Planen Sie die Bearbeitung der nächsten Lektion so, dass Sie in der Zwischenzeit die Gelegenheit haben, Ihre beste Lösung zum 6-Schritte-Plan auszuprobieren.

Ich werde die nächste Lektion am	den	um ca.	
Donnerstag	30.	18:00	Uhr bearbeiten.

Fit im Stress - Lektion 2

Wie ist es Ihnen ergangen?

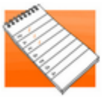

In der vorigen Lektion haben Sie das Programm kennengelernt, grundlegende Informationen zum Thema Stress erhalten, sich Ihre persönlichen Kraftgeber bewusst gemacht, die Stress-Analyse begonnen und sich mit dem Thema Motivation beschäftigt.

War es Ihnen möglich, die Übungen im Alltag zur Lektion 1 (die persönlichen Kraftgeber, die Stress-Analyse und das Stresstagebuch) umzusetzen?

- (•) Ja
- ( ) Teilweise
- ( ) Nein

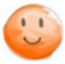

Das ist klasse! Wenn Sie möchten, können Sie hier einmal berichten, wie es Ihnen ergangen ist und was Sie als besonders hilfreich empfunden haben.
